# Vascularized Co-Culture Clusteroids of Primary Endothelial and Hep-G2 Cells Based on Aqueous Two-Phase Pickering Emulsions

**DOI:** 10.3390/bioengineering9030126

**Published:** 2022-03-21

**Authors:** Anheng Wang, Leigh A. Madden, Vesselin N. Paunov

**Affiliations:** 1 Department of Chemistry, University of Hull, Hull HU6 7RX, UK; a.wang-2019@hull.ac.uk; 2 Department of Biomedical Sciences, University of Hull, Hull HU6 7RX, UK; l.a.madden@hull.ac.uk; 3 Department of Chemistry, Nazarbayev University, Nur-Sultan 010000, Kazakhstan

**Keywords:** co-culture, angiogenesis, water-in-water Pickering emulsions, 3D cell culture

## Abstract

Three-dimensional cell culture has been extensively involved in biomedical applications due to its high availability and relatively mature biochemical properties. However, single 3D cell culture models based on hydrogel or various scaffolds do not meet the more in-depth requirements of in vitro models. The necrotic core formation inhibits the utilization of the 3D cell culture ex vivo as oxygen permeation is impaired in the absence of blood vessels. We report a simple method to facilitate the formation of angiogenic HUVEC (human umbilical vein endothelial cells) and Hep-G2 (hepatocyte carcinoma model) co-culture 3D clusteroids in a water-in-water (*w*/*w*) Pickering emulsions template which can overcome this limitation. This method enabled us to manipulate the cells proportion in order to achieve the optimal condition for stimulating the production of various angiogenic protein markers in the co-cultured clusteroids. The HUVEC cells respond to the presence of Hep-G2 cells and their byproducts by forming endothelial cell sprouts in Matrigel without the exogenous addition of vascular endothelial growth factor (VEGF) or other angiogenesis inducers. This culture method can be easily replicated to produce other types of cell co-culture spheroids. The *w*/*w* Pickering emulsion template can facilitate the fabrication of 3D co-culture models to a great extent and be further utilized in drug testing and tissue engineering applications.

## 1. Introduction

Three-dimensional cell culture models are achieved using non-adhesive flasks, hydrogel, or microfluidic devices to drive the cells to aggregate into a densely packed spherical form [1,2,3]. These 3D cell culture models have been extensively used in drug testing application since they were introduced in the last century [4,5]. Compared to the commonly used animal models, these in vitro 3D models are free of sophisticated operations and strict ethical review [6,7]. The high availability and precise mimicry of real-life environments has led to a great deal of interest in this area. Drug testing and drug release kinetics on 3D spheroids and organoids can be considered a pre-clinical assessment [8,9,10]. Organoids models have become popular since the first discovery of cancer organoids in vitro, enabling scientists to develop organoids biobanks based on patients’ biopsy samples. Another downstream application of 3D cell culture is utilizing the spheroids to construct artificial organs in vitro using additive manufacturing [11,12]. There have been several reports demonstrating the usage of spheroids in repairing or replacing damaged skin or bone. The application of 3D cell culture still has its Achilles’ heel: necrotic core formation. The formation of the necrotic core in 3D non-endothelial cell spheroids is ineluctable due to the spatiotemporal gradients of chemicals and oxygen in spheroids’ proliferation [13,14].

There are still serious challenges for the pre-clinical use of 3D spheroids including uniformity, reproducibility, yield, and assessment method [15]. Herein, pre-vascularization of the spheroids before applying them is a crucial step for longer term experiments. Several growth factors, primarily VEGF, are responsible for angiogenesis in vivo [16,17]. These factors firstly activate the transformation of endothelial cells from a resting stage into activated tip cells under the control of Notch signaling [18]. The release of metalloproteinases (MMPs) follows in order to stimulate the endothelial cells degrading and migrating into the surrounding tissue or extracellular matrix (ECM) in vivo [19,20]. The endothelial stalk cells result in the formation of capillary buds and sprouts due to the angiogenic stimulus [21]. Finally, the sprouts form a lumen and interconnect with each other to new blood-perfused micro-vessels. Lumen-like structures have been observed in endothelial cell and cancer cell co-culture models [22,23,24]. A cancer cell line can work as an endothelial growth factor pump to stimulate the transformation of endothelial cells into capillary buds and sprouts via the release of stimulatory factors. These co-culture spheroids are commonly based on microfluidic devices which require high labor costs and specialized equipment. The scaffold or non-adhesive flasks-based technology suffers in achieving a high yield of spheroids. A convenient production method that can generate co-culture 3D cell models on a larger scale with low cost is required. Here, we report that an aqueous two-phase system (ATPS) in the form of a water-in-water Pickering emulsion template, previously developed, can be potentially adapted to facilitate a high throughput fabrication of co-cultured vascularized clusteroids [25,26,27]. The use of Pickering water-in-water emulsions is attracting increased attention after the first report from Poortinga et al. [28]. Several particles including dopamine-based particles, cellulose, and protein particles have been studied [29,30,31]. The low interfacial tension of this emulsion system makes it an ideal model for biomedical and food applications [32].

**Figure 1:**
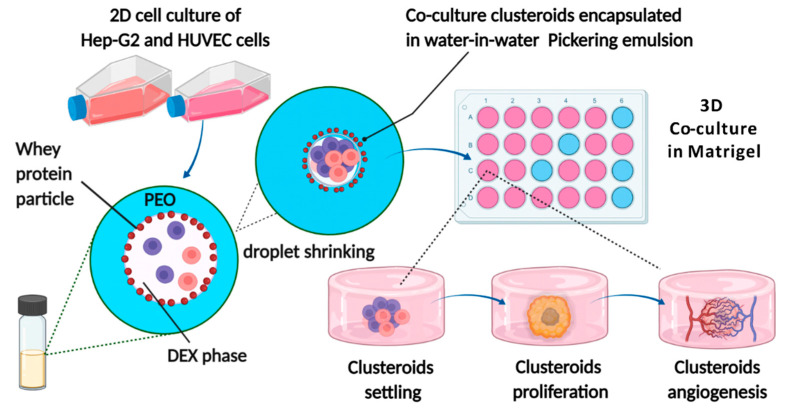
Schematic illustration of the HUVEC and Hep-G2 co-culture clusteroids in the *w*/*w* Pickering emulsion template and clusteroids angiogenesis. Created with BioRender.com.

In the present study, we demonstrated how human umbilical vein endothelial cells (HUVEC) could be used to vascularize Hep-G2 cells upon co-culturing them using the *w*/*w* Pickering emulsion template. Hep-G2 cells could easily be swapped to any other cell line or patient biopsy sample to study vascularization in vitro (Figure 1). Ideally, co-cultured clusteroids use any two cell types based on the desired functionality of the 3D model. The 3D co-cultured clusteroids obtained in our study showed a significant increase in angiogenesis compared to the single cell type clusteroids. We found that the optimal cell ratio of the co-culture is Hep-G2:HUVEC = 5:1, where the clusteroids spontaneously sprouted into the Matrigel. This facile platform would greatly reduce the threshold of generating co-culture spheroids in a laboratory for various biomedical and tissue engineering applications.

## 2. Materials and Methods

### 2.1. Materials

CFSE far-red, CFSE green fluorescence dye, easYFlasks, and NUNC cell culture 24-well plates were purchased from Thermo Fisher Scientific (Loughborough, UK). Dextran (DEX) (MW 500 kDa) was purchased from Alfa Aesar (Heysham, Lancashire, UK), sodium chloride (99.8%) and calcium chloride were sourced from Eagle’s Modified Eagle Medium, and Trypsin-EDTA were sourced from Gibco (Loughborough, UK). Fetal bovine serum (FBS) was sourced from Labtech (Heathfield, UK) and trypsin-EDTA was purchased from Lonza (UK). Endothelial cell culture medium was purchased from ATCC, HUVEC cell line was sourced from Promocell (Lutterworth, Leicestershire, UK), and Hep-G2 cell line was purchased from ECACC cell collection (Salisbury, UK). The MMP-2 ELISA kit was purchased from GE healthcare (Amersham, UK), the IGFBP, VEGF, IL-8, and HIF1-α DuoSet ELISA kits and angiogenesis array kit (ARY007) were all purchased from Bio-Techne (Abingdon, UK). The 2 wt% gelatin suspension was sourced from Sigma Aldrich (Gillingham, UK) and Matrigel was purchased from Corning (Flintshire, UK). Whey protein was sourced from No.1 Supplements (Suffolk, UK). Deionized water was purified by using MilliQ reverse osmosis water purification system (Merck, Millipore, Burlington, MA, USA). All the other chemicals were of analytical grade.

### 2.2. Methods

#### 2.2.1. HUVEC and Hep-G2 Monolayer Cell Culture

HUVEC and Hep-G2 cell lines were cultured in complete endothelial cell medium (ATCC, Manassas, VA, USA) and EMEM medium supplemented with 10% FBS, respectively. Hep-G2 cells were cultured in T75 easYFlask at 37 °C with 5% CO_2_. HUVEC cells were cultured in a T75 easYFlask (ThermoFisher Scientific, Waltham, MA, USA) precoated with 1 wt% gelatin suspension. The cells were passaged after they reached 80% confluency using 0.25 wt% trypsin solution and passaged in a 1:4 ratio for both cell lines.

#### 2.2.2. HUVEC and Hep-G2 3D Clusteroids Culture

The fabrication of co-culture clusteroids is based on the aqueous two-phase system, developed as a water-in-water Pickering emulsion. The clusteroids preparation method used here was adapted from Wang et al. [27] and developed earlier by Das et al. [33] and Celik et al. [34]. Briefly, freshly prepared 22 wt% PEO (poly-ethylene oxide) and 11 wt% Dextran (DEX) solutions were sterilized by autoclaving (121 °C, 15 min). An equal volume of the 22 wt% PEO solution and 1 wt% heat-treated whey protein particle suspension (WPP) was thoroughly mixed using magnetic agitation to prepare a 11 wt% PEO-0.5 wt%WPP solution. WPP was then either UV sterilized or 0.45 μm filter-sterilized to avoid contamination. The 11 wt% PEO-0.5 wt%WPP solution was further mixed with equal volume of medium (EMEM or endothelial cell medium) to prepare the 5.5 wt%/0.25 wt% WPP/Medium solution. The 11 wt% Dextran (DEX) solution was also diluted using medium to generate 5.5 wt% DEX/Medium solution. The cells were harvested by centrifugation, resuspended in DEX/culture medium and then mixed with PEO/culture medium solution using a syringe and needle. The generated *w*/*w* emulsion droplets were then shrunk by adding PEO solution of higher concentration in culture medium overnight to generate co-culture cell clusteroids. The initial cell ratio and concentration was adjusted using Trypan Blue staining and a hemocytometer.

#### 2.2.3. Long-Term Growth of the Co-Culture Clusteroids in Matrigel

The support of an extracellular matrix (ECM) is essential in the formation of endothelial networking, especially for the cell sprouts in the angiogenesis process in vitro. The most commonly used ECM for HUVEC is Matrigel. Here, we utilized Matrigel to support the proliferation of clusteroids. The Matrigel was kept frozen in ice to avoid polymerization before it was used. Matrigel was diluted in media (50/50 *v*/*v* EMEM/endothelial cell growth medium) to 5 mg mL^−1^ before use. After the formation of clusteroids, the clusteroids were centrifugated at 300× *g* for 4 min to collect them as a pellet. The pellet was then resuspended in 500 μL Matrigel and transferred to a 24-well plate. The initial cell number of the clusteroids in Matrigel was 1 × 10^5^ mL^−1^. Matrigel was allowed to polymerize in the incubator at 37 °C for 30 min. The culture was then topped up with 500 μL complete medium (supplemented with 10% FBS) and incubated at 37 °C with 5% CO_2_. For the individual cell clusteroids, either endothelial cell medium or EMEM medium was used. The 50/50 (*v*/*v*) endothelial cell medium/EMEM medium was used for the co-culture clusteroids. The medium was replaced every 2 days.

#### 2.2.4. Bright-Field, Fluorescence, and Confocal Microscopy Observations

Bright-field optical and fluorescence microscopy was employed to characterize the microstructure of the emulsions and encapsulated cell clusters using the Olympus BX-51 Fluorescence microscope (OLYMPUS UK, Southend-on-Sea, UK). CFSE and CFSE far-red were used as the fluorescence dyes to stain the Hep-G2 and HUVEC cells, respectively. These two dyes were also used for the longer-term tracking of clusteroids proliferation. The clusteroids were further characterized using the confocal laser scanning microscope (CLSM, Zeiss LSM710).

#### 2.2.5. Spheroid Sprouts Analysis

The generation of sprouts requires a low-serum medium. For this purpose, the co-cultured clusteroids medium was changed to complete endothelial medium/EMEM medium supplemented with only 2% *v*/*v* FBS. The clusteroids were also embedded in Matrigel with an initial cell concentration of 1 × 10^5^ cells mL^−1^ in 24 well plates. The cultures were topped up with 500 μL complete medium (supplemented with 2% *v*/*v* FBS) and incubated at 37 °C with 5% CO_2_. The clusteroids sprouts were analyzed by the WimSprout assay (Wimasis Image Analysis, Córdoba, Spain) and the size of the 20 longest sprouts from the clusteroids were measured to estimate the degree of angiogenesis.

#### 2.2.6. HIF1-α, MMP-2, IGFBP, and VEGF ELISA 

A 500 μL aliquot of the 3D clusteroids culture’s supernatant was collected for testing at different days of culture (on days 1, 7, 14, and 21). MMP-2 was quantified following the manufacturer’s instructions. The remaining ELISA kits were developmental antibody pairs (IL-8, IGFBP, HIF1-α, and VEGF) used with appropriate ancillary reagents. For these, 100 μL of specified capture antibody was added to each well of the 96-well plates overnight at room temperature to coat the well. The plates were then rinsed three times with wash buffer using an automatic plate washer. Plates were blocked by adding 300 μL of reagent diluent to each well and incubated at room temperature for a minimum of 1 h. A 100 μL aliquot of the clusteroids supernatant was added to each well and incubated for 2 h. A 100 μL aliquot of the biotinylated detection antibody then Streptavidin-HRP and substrate solution were added in order with incubation and washing between each step. Finally, an aliquot of 50 μL stop solution was added last to each well. The optical density of each well was determined immediately with a wavelength of 450 nm using a Synergy HT (Agilent Technologies LDA UK Limited, Stockport, Cheshire, UK) microplate reader.

#### 2.2.7. SEM Imaging of the Clusteroids

The clusteroids were released from Matrigel after 21 days of culture using collagenase, which enzymatically degrades the gel. The clusteroids were then centrifuged and rinsed twice with PBS (phosphate-buffered saline) to remove the excess of hydrogel and the medium. The clusteroids were then fixed in a 1 wt% glutaraldehyde solution for 2 h at ambient temperature, washed with deionized water, then air-dried overnight before being imaged with Zeiss smart SEM software (Zeiss Evo-60 SEM., Oberkochen, Germany).

#### 2.2.8. Angiogenesis Array

The angiogenesis-related protein markers produced in the 3D co-culture clusteroids were analyzed using the proteome profiler angiogenesis array kit according to the manufacturer’s instructions. A 1 mL aliquot of the conditioned media was obtained from the 3D clusteroids (individual Hep-G2, individual HUVEC, and co-cultured clusteroids (Hep-G2: HUVEC = 5:1) embedded in the 5 mg mL^−1^ Matrigel).

Array membranes were first blotted for 1 h. To reach the optimal sensitivity, the array was incubated overnight with the 1 mL of conditioned media at room temperature with gentle shaking. The membranes were carefully submerged in a wash buffer for 10 min with three repeats to remove non-attached proteins. The array antibody cocktail and a horseradish peroxidase-conjugated streptavidin antibody were incubated with the membranes for another 12 h. Membranes were then mixed with 1× detection buffers. The membranes were then imaged using a Bio-Rad image system exposed for 200 s set at chemiluminescence blot (ATTO, WSE 6100 LuminoGraph I, Yuseong-gu, Daejeon, Korea). The ChemiBlot intensity was calculated using GelAnalyzer 19.1 (www.gelanalyzer.com, accessed 14 November 2021). Relative expression levels in each group were determined using the algorithm stated in the manufacturer’s protocol.

#### 2.2.9. Statistical Analysis

In the experimental sections, three independent experiments were carried out to present the mean of experiments ± SD (Standard Deviation). The comparison between two groups were done using 2-tailed independent-sample t-tests. Statistical significance was defined as *p* < 0.05 or *p* < 0.01.

## 3. Results and Discussion

### 3.1. Clusteroids Culture in the w/w Pickering Emulsion Template

**Figure 2:**
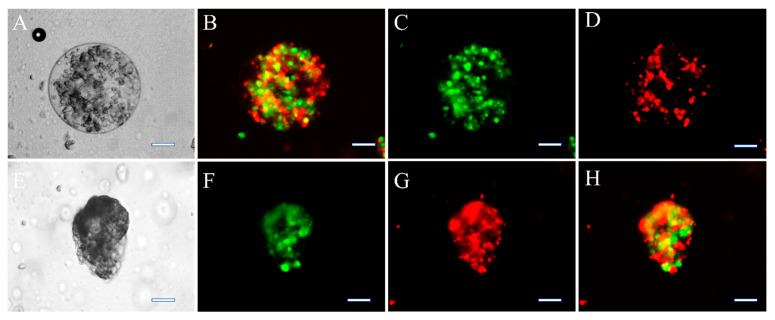
Bright-field (**A**,**E**) and fluorescence microscope observation of mixed Hep-G2 and HUVEC (cell ratio = 5:1) cells encapsulated by 5.5 wt% DEX in 5.5 wt% PEO emulsions before (**A**–**D**) and after shrinking by 11 wt% PEO (**E**–**H**) set at FITC (**B**,**F**), CFSE far-red set at TRITC (**C**,**G**), and dual fluorescence FITC and TRITC channels (**D**,**H**). Clusteroids in the emulsions (**A**–**D**) and clusteroids taken out from the emulsions (**E**–**H**) were taken for imaging immediately after their formation. The Hep-G2 cells and HUVEC cells were pre-stained with CFSE and CFSE far-red, respectively. The bar is 50 μm.

The cell encapsulation and formation of clusteroids was achieved using a *w*/*w* DEX/PEO Pickering emulsion stabilized by biocompatible WPP particles as template. Other studies have shown that cells in DEX/PEO ATPS preferentially accumulate inside the DEX phase [35,36]. The cell concentration and the volume fraction of the two aqueous phases (PEO and DEX) are important in generating the clusteroids [25]. The schematic illustration of how we utilized the *w*/*w* emulsion template is outlined in Figure 1. Here, we set the initial cell concentration to 10^6^ cells mL^−1^ (Figure S1 in Supplementary Materials). To generate clusteroids of larger size, the cell number can be increased while keeping the other conditions equal. After the formation of the *w*/*w* DEX/PEO emulsion with the cells, it was immediately topped up to a PEO solution of higher concentration to osmotically shrink the DEX drops with the cells into clusteroids. The DEX droplets shrink due to the redistribution of water to restore the osmotic equilibrium between the DEX and PEO phases. The interfacial tension forces then compact the cells where they adhere to each other to form clusteroids (Figure 2E,F). Note that the mass density of the cells is lower than the 5.5 wt% DEX phase and higher than the PEO phase at any concentration [37]. The encapsulation efficiency of the cells was first observed using fluorescence microscopy.

**Figure 3:**
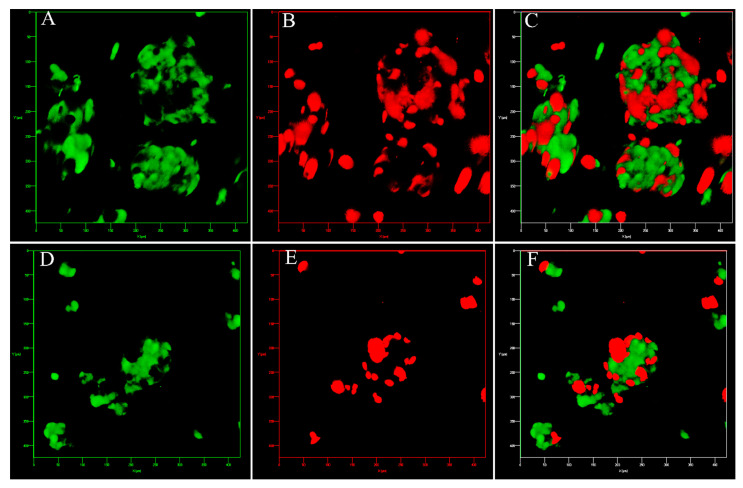
Three-dimensional Z-stacked image of co-cultured Hep-G2 and HUVEC clusteroids at a cell ratio of (**A**–**C**) 1:1 and (**D**–**F**) 5:1 set at different fluorescence channels: FITC (**A**,**D**), CFSE far-red (**B**,**E**), and dual fluorescence (**C**,**F**). The box size is 400 μm × 400 μm.

**Figure 4:**
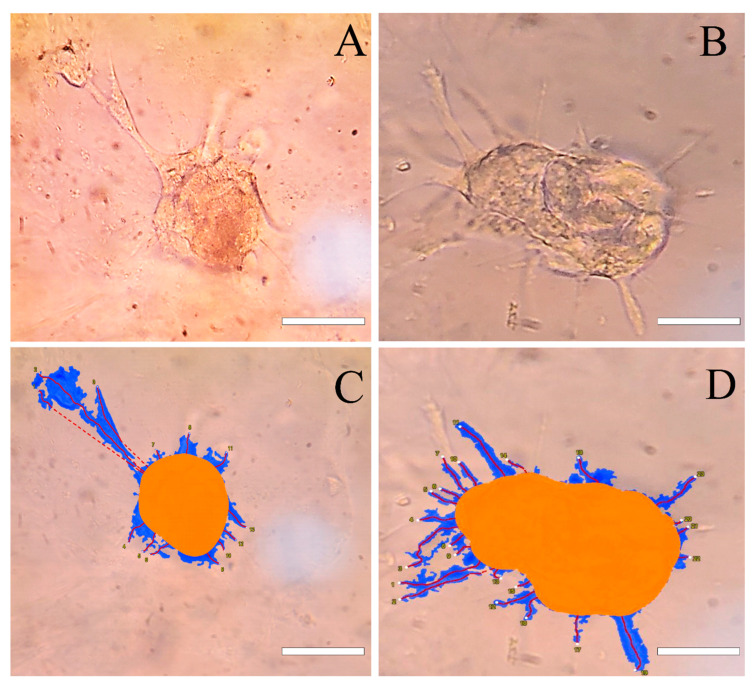
(**A**–**D**): Bright-field observation of the co-culture clusteroids sprouting after 7 days of culture with a cell ratio of 5:1 (Hep-G2:HUVEC) in 5 mg mL^−1^ Matrigel. The corresponding Wimsprouts analysis of (**A**,**B**) is shown in (**C**,**D**). The bar is 50 μm.

**Figure 5:**
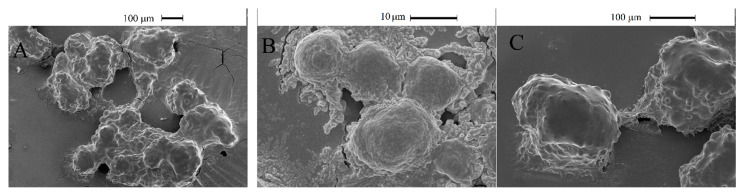
SEM observation of the: (**A**) individual Hep-G2 clusteroids, (**B**) individual HUVEC clusteroids, and (**C**) co-culture of Hep-G2 and HUVEC clusteroids at a cell ratio of 5:1. The cells kept growing in the Matrigel before the gels were degraded.

The Hep-G2 and the HUVEC cells were labelled with CFSE (green) and CFSE far-red (red), respectively. Figure 2A–D shows the cell’s localization within the *w*/*w* emulsion which confirms that most of the cells resided in the DEX phase. The clusteroids that were formed after the DEX drops contraction by the higher concentrated PEO solution are shown in Figure 2E–H. It can be seen that the extra space between the cells was diminished and the cells were allowed to closely contact each other. The two kinds of cells were evenly distributed within the formed clusteroids. The formation of the individual clusteroids (Hep-G2 and HUVEC) is shown in Figure S2 in Supplementary Materials. These results show the high efficiency of the *w*/*w* Pickering emulsion system in preparation of 3D co-culture cell clusteroids. We further employed confocal microscopy to observe how the cells arranged within the clusteroids in 3D (Figure 3). The formed mixed clusteroids were centrifugated and resuspended in a complete medium for confocal microscopy observations. After the clusteroids were taken out from the emulsion template, their structure maintained its integrity (Figure S2 in Supplementary Materials). We examined the structure of the clusteroids at two different cell ratios (Hep-G2:HUVEC = 1:1 and Hep-G2:HUVEC = 5:1). The two cell ratios did not appear to impact the distribution of the cells in the mixed clusteroids after a long period of culturing. Observation of the Hep-G2 clusteroids showed a smooth layered surface and a strong tendency towards fusion (Figure 4A and Figure S3 in Supplementary Materials). The HUVEC cell clusteroids were surrounded by structures that may indicate neovascularization (Figure 4B). The tail-like structure was observed in the co-cultured clusteroids (Figure 4C). The formation of sprouts was not possible to directly visualize by SEM images of the same samples (Figure 5).

### 3.2. Production of Angiogenic Factors by the Co-Cultured Clusteroids 

HUVEC and Hep-G2 cell co-culture is one of the most studied models using various methods including microfluidic devices, scaffolds, and hydrogels [38,39,40]. To obtain a better understanding of which protein markers related to angiogenesis were produced in the clusteroids, the supernatant of these clusteroids embedded in the Matrigel was collected for analysis via a proteome angiogenesis array. The production of these markers in the co-cultured (HUVEC and Hep-G2) clusteroids and the individual Hep-G2 and HUVEC clusteroids was compared.

**Figure 6:**
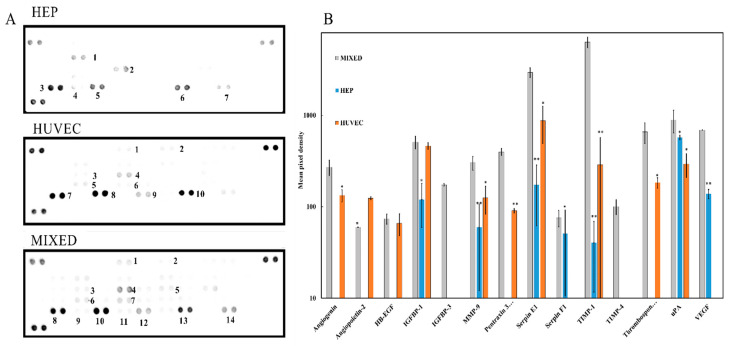
(**A**) Angiogenesis array membrane of individual Hep-G2 clusteroids, HUVEC clusteroids, and the co-culture Hep-G2 and HUVEC clusteroids at a Hep-G2 to HUVEC cell ratio of 5:1 after 21 days of proliferation in the Matrigel. The initial total cell number was 1 × 10^6^ mL^−1^. (**B**) Angiogenesis-related protein production in the individual Hep-G2 clusteroids, HUVEC clusteroids, and the co-culture Hep-G2 and HUVEC clusteroids. Data were plotted as mean ± standard deviation of at least three independent experiments. Statistically significant differences between each region are denoted by * (*p* < 0.05) or ** (*p* < 0.01).

The angiogenesis array containing an antibody cocktail can detect 55 different human angiogenesis-related proteins simultaneously. Captured proteins are visualized using chemiluminescent detection reagents. The signal produced is proportional to the amount of bound analyte. The intensity of different detected proteins is compared by their relative density against the controls on the corner of each membrane. Figure 6A shows that the overall production of angiogenesis markers produced by either HUVEC or Hep-G2 increased. The effect of these produced proteins on angiogenesis is detailed in Table S1 in Supplementary Materials. Specifically, the levels of angiogenin, MMP-9, uPA, IGFBP, and VEGF were higher in the co-cultured HUVEC and Hep-G2 clusteroids when compared to the two individual cell type clusteroids (Figure 6A,B). The production of IGFBP-3 and TIMP-4 was only detectable in the co-cultured HUVEC and Hep-G2 clusteroids, which both work as angiogenesis inducers. TIMP-1 has a negative effect on angiogenesis which inhibits the production of tissue plasminogen activator (tPA) and urokinase (uPA) [41]. The co-efficiency of the TIMP and uPA families balances the angiogenesis process, and TIMP-1 also blocks the production of matrix metalloproteinases (MMPs) [42]. 

Another important finding from our study is that the HUVEC cell clusteroids alone were not able to produce VEGF. The results indicate that the co-cultured pattern strongly increases the secretion of angiogenesis-related proteins.

Some protein production was only triggered when the two types of cells (HUVEC and Hep-G2) were co-cultured together in 3D form. The results of the angiogenesis array were able to provide information about the secretion of these angiogenesis proteins. To understand how these protein markers were produced over the 21 days, we performed several ELISA assays including VEGF, IGFBP-1, MMP-2, and HIF1-α. VEGF is the most commonly used angiogenesis-related cytokine for inducing endothelial cell vascularization in vitro endothelial [43]. We tested the VEGF production of the three kinds of cell clusteroids using their supernatant. 

**Figure 7:**
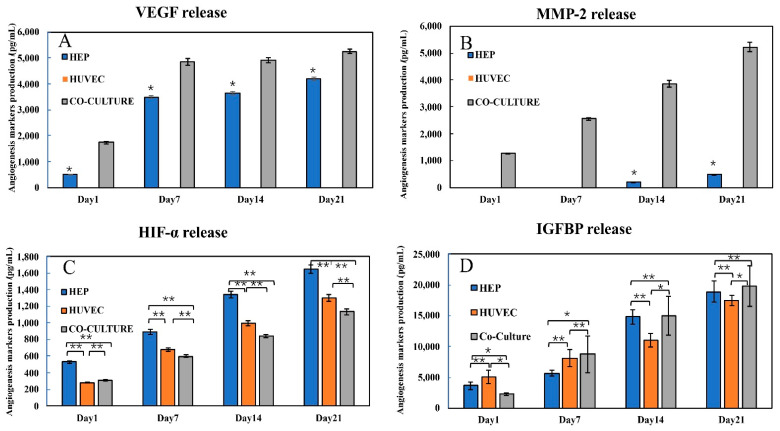
(**A**): VEGF; (**B**): MMP-2; (**C**): HIF1-α; and (**D**): IGFBP-1. Production of the individual Hep-G2 clusteroids, HUVEC clusteroids, and co-culture clusteroids at a cell ratio of 5:1 (Hep-G2: HUVEC) during 21 days of proliferation in 5 mg mL^−1^ Matrigel. The initial total cell number was 1 × 10^6^ mL^−1^ for all three sets. Data were plotted as mean ± standard deviation of at least three independent experiments. Statistically significant differences between each region are denoted by * (*p* < 0.05) or ** (*p* < 0.01).

It can be seen from Figure 7A that while VEGF was not detected in the HUVEC clusteroids, the co-cultured (Hep-G2 and HUVEC) clusteroids produced about 50% more VEGF than the individual Hep-G2 clusteroids. This result corroborated the result obtained from the proteome angiogenesis array. Another important marker related to endothelial cell sprouting is the MMP family of proteins that breaks down the extracellular matrix (ECM). The production of MMP-2 in the individual HUVEC cells was not detectable, indicating that without the exogenous VEGF induction, the HUVEC would not be able to sprout into the ECM (Figure 7B). MMP-2 production in the Hep-G2 was very low after 14 days of culture. The co-culture models produced five times higher amount of the MMP-2 when compared to the Hep-G2 cell clusteroids, which indicates its effect on possible sprouting of the co-cultured clusteroids. 

MMP-2 and VEGF production within the 21 days was a clear sign of angiogenesis occurrence in the co-cultured clusteroids. HIF1-α is a transcription factor that responds to decreases in available oxygen in the cellular environment, or hypoxia. This substance can work as a reverse indicator of the vascularization state of the clusteroids. The results in Figure 7C demonstrate that the co-culture clusteroids produced the lowest amount of HIF1-α from day 1 to 21. The proliferation of the three kinds of clusteroids inevitably makes it difficult for oxygen and nutrients to reach the core cells. Thus, the production level of HIF1-α is increased. Insulin-like growth factors (IGFs) are proteins with high sequence similarity to insulin. IGFs are part of a complex system that cells use to communicate with their physiologic environment. IGF-1 and IGF-2 are regulated by a family of proteins known as IGF-binding proteins. These proteins help to modulate IGF action in complex ways that involve both inhibiting IGF action by preventing binding to the IGF-1 receptor and promoting IGF action possibly through aiding delivery to the receptor and increasing the IGF half-life. The IGFBP production in the three types of clusteroids was similar to the result of the angiogenesis array. (Figure 7D). The secretion of this IGFBP was also the highest in the co-cultured clusteroids after seven days of the culture. The HUVEC produced the most IGFBP in the first seven days. This can be attributed to the initial self-sorting stage of the co-cultured clusteroids. 

### 3.3. Endothelial Cell Sprouting 

The spheroid-based sprouting assay is a well-established and robust method to study the influence of genetic alterations or pharmacological compounds on capillary-like tube formation of primary cultured endothelial cells [44,45]. The most widely used ECM for endothelial cell lines is Matrigel. Matrigel is the trade name for the solubilized basement membrane matrix secreted by Engelbreth-Holm-Swarm (EHS) mouse sarcoma cells (produced by Corning Life Sciences, Flintshire, UK). Here, we performed co-cultured Hep-G2 and HUVEC clusteroids sprouting in 5 mg mL^−1^ Matrigel. In contrast with general HUVEC spheroids sprouting assays, no exogenous VEGF or EGF was needed. Hep-G2 cells were expected to work as a VEGF pump for HUVEC sprouting. The length of the clusteroids sprouting was an indicator of the angiogenesis degree of the 3D cell culture.

In our test, without the addition of any VEGF, sprouting could be easily observed in Hep-G2 and HUVEC co-cultured clusteroids with a cell ratio of 5:1 after seven days of culture (Figure 4). The individual Hep-G2 cells and HUVEC cells were not able to sprout into the ECM. (Figure S4 in Supplementary Materials). This suggests that unique cell-cell interaction is needed for the observed sprouting behavior. The results obtained from the ELISA kit and angiogenesis array kit might explain the reason for this outcome. The length of each sprout is given in Figure S5 in Supplementary Materials. HUVEC cells were not shown to produce VEGF, which may stimulate endothelial cell sprouting. Hep-G2 cells produce a high amount of VEGF as it is a carcinoma. The combination of the endothelial cells and carcinoma cell lines can therefore induce angiogenesis formation and sprouting. MMP-2 production can also indicate that the ECM is being broken down by HUVEC cells. Only a limited amount of MMP-2 was secreted by the Hep-G2 clusteroids. MMP-2 is a crucial protein when the clusteroids sprouts into the ECM [46]. The production of MMP-2 in the co-cultured clusteroids was significantly enhanced, greater by about five-fold than the individual Hep-G2 clusteroids after 21 days of culture (Figure 7B). Previous reports of culturing carcinoma and endothelial cell spheroids observed similar results without the addition of VEGF, i.e., the carcinoma cells would stimulate co-cultured spheroids to sprout [45]. The observation of the SEM also indicated the existence of several tail-like structures in the co-cultured clusteroids (Figure 5C) that was not detected in the individual clusteroids (Figure 5A,B). The current work aims to develop a reliable new protocol for the in vitro 3D cell co-culture model with angiogenesis potential. This latest model may have broad implications in drug testing applications, particularly those that attempt to prevent new blood vessel formation. Anti- and pro-angiogenic treatments may be applied on this model to reveal their efficiency. The clusteroids can be further characterized using immunohistochemistry to show the integrity and structural change during treatments.

## 4. Conclusions

We report a 3D platform of co-culture cell clusteroids of hepatic tumor cells and primary endothelial cells which showed in vitro behavior consistent with its reported in vivo behavior. By adapting the cell ratio to 1:2 (Hep-G2:HUVEC), which was close to that observed in vivo environment, the co-cultured clusteroids obtained from the ATPS could sprout into Matrigel. Various angiogenesis protein production was boosted in the co-culture clusteroids compared to the individual cell type. This approach can easily be handled with no special instruments or expensive consumables. The cells can be swapped to any two cell types for distinct purposes except 3D cell vascularization. This model can be further utilized for the study of drug toxicology amongst further tissue engineering applications.

## Data Availability

Not applicable.

## Supplementary Materials

The following supporting information can be downloaded at: https://www.mdpi.com/article/10.3390/bioengineering9030126/s1, Figure S1: Different cell concentration of mixed HUVEC and Hep-G2 cells in 5.5 wt% DEX/5.5 wt% PEO *w*/*w* Pickering emulsion with different cell concentration before shrinking: (A) 1 × 10^3^ mL^−1^; (B) 1 × 10^4^ mL^−1^; (C) 1 × 10^5^ mL^−1^; and (D) 1 × 10^6^ mL^−1^. The bar is 100 μm for A,B and 50 μm for C,D. Figure S2: Bright-field observation (A) and fluorescence microscopy observation (B) of the freshly collected Hep-G2 and HUVEC co-culture clusteroids at a cell ratio of 5:1. The bar is 100 μm. Figure S3: Angiogenesis-related protein production in the three types of clusteroids (HUVEC, Hep-G2, and mixed Hep-G2 and HUVEC) corresponding to Figure 4A. Figure S4: Bright-field observation of the individual Hep-G2: HUVEC clusteroids in 5 mg mL^−1^ Matrigel after 21 days of culture in low-serum medium. The bar is 50 μm. Figure S5: The length of the sprouts in Table S1. Table S1. The effect of the angiogenesis-related genes on the vascularization process. (+) represents that they are pro-angiogenesis.
