# Supplementary material for: Vascularized Co-Culture Clusteroids of Primary Endothelial and Hep-G2 Cells Based on Aqueous Two-Phase Pickering Emulsions
Source: Bioengineering (Basel). 2022 Mar 21;9(3):126. doi: 10.3390/bioengineering9030126 (PMC8945860; doi:10.3390/bioengineering9030126)
Supplement: Supplementary file 1 [file bioengineering-09-00126-s001.zip › bioengineering-1575969-supplementary.pdf]

bioengineering

MDPI

Supplementary Materials

Vascularized Co-Culture Clusteroids of Primary Endothelial and Hep-G2 Cells Based on Aqueous Two-Phase Pickering Emulsions

Anheng Wang [1], Leigh A. Madden [2] and Vesselin N. Paunov [3,*]

[1] Department of Chemistry, University of Hull, Hull HU67RX, UK; a.wang-2019@hull.ac.uk
[2] Department of Biomedical Sciences, University of Hull, Hull HU67RX, UK; l.a.madden@hull.ac.uk
[3] Department of Chemistry, Nazarbayev University, Kabanbay Batyr Avenue 53, Nur-Sultan 010000, Kazakhstan
* Correspondence: vesselin.paunov@nu.edu.kz

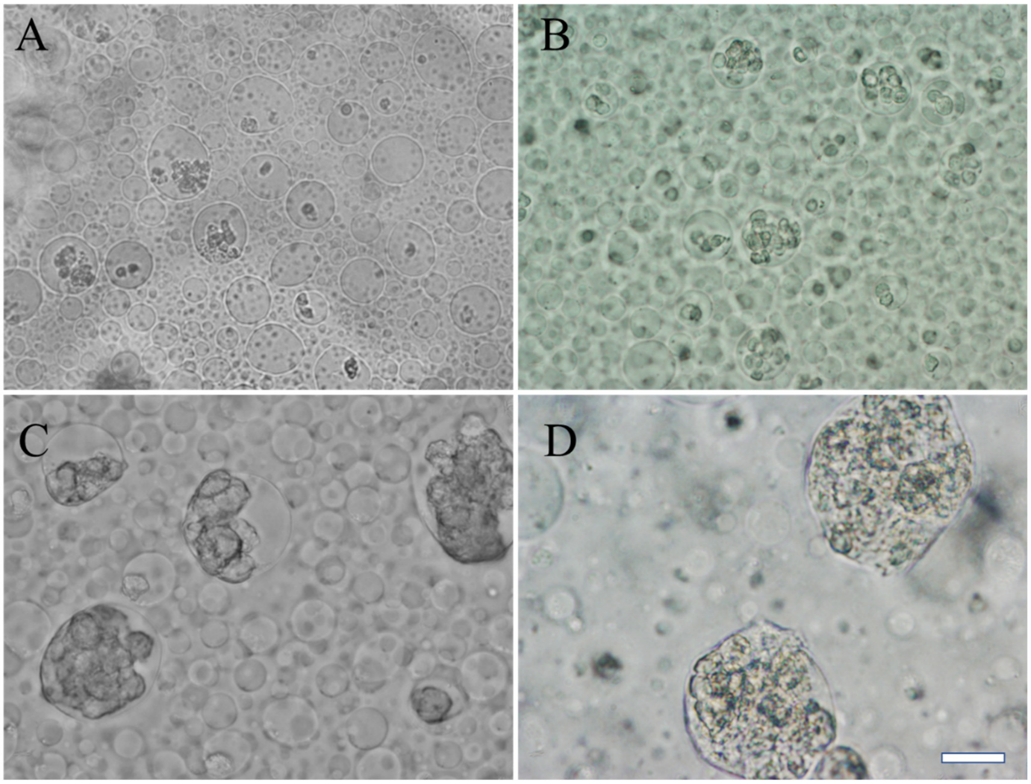

Figure S1. Different cell concentration of mixed HUVEC and Hep-G2 cells in 5.5 wt% DEX/5.5 wt% PEO w/w Pickering emulsion with different cell cocentration before shrinking: (**A**) 1×10^3 mL^{-1}; (**B**) 1×10^4 mL^{-1}; (**C**) 1×10^5 mL^{-1}; and (**D**) 1×10^6 mL^{-1}. The bar is 100 μm for A,B and 50 μm for C,D.

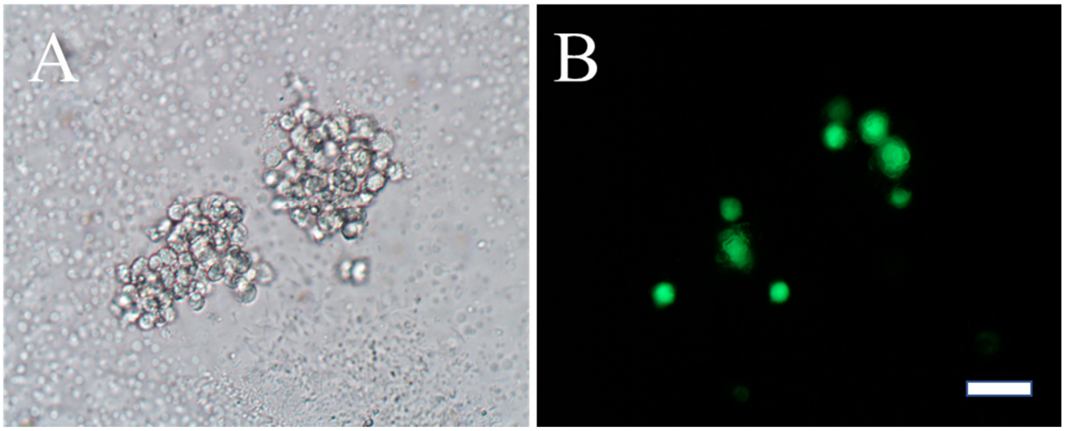

Figure S2. Bright-field observation (**A**) and fluorescence microscopy observation (**B**) of the freshly collected Hep-g2 and HUVEC co-culture clusteroids at a cell ratio of 5:1. The bar is 100 μm.

MIXED	
1	Angiogenin
2	Angiopoietin-2
3	HB-EGF
4	IGFBP-1
5	IGFBP-3
6	MMP-9
7	Pentraxin 3 (PTX3)
8	Serpin E1
9	Serpin F1
10	TIMP-1
11	TIMP-4
12	Thrombospondin-1
13	uPA
14	VEGF

HUVEC	
1	Angiogenin
2	Angiopoietin-2
3	HB-EGF
4	IGFBP-1
5	MMP-9
6	Pentraxin 3 (PTX3)
7	Serpin E1
8	TIMP-1
9	Thrombospondin-1
10	uPA

HEP	
1	IGFBP-1
2	MMP-9
3	Serpin E1
4	Serpin F1
5	TIMP-1
6	uPA
7	VEGF

Figure S3. Angiogenesis-related protein production in the three types of clusteroids (HUVEC, Hep-G2, and mixed Hep-G2 and HUVEC) corresponding to Figure 4A.

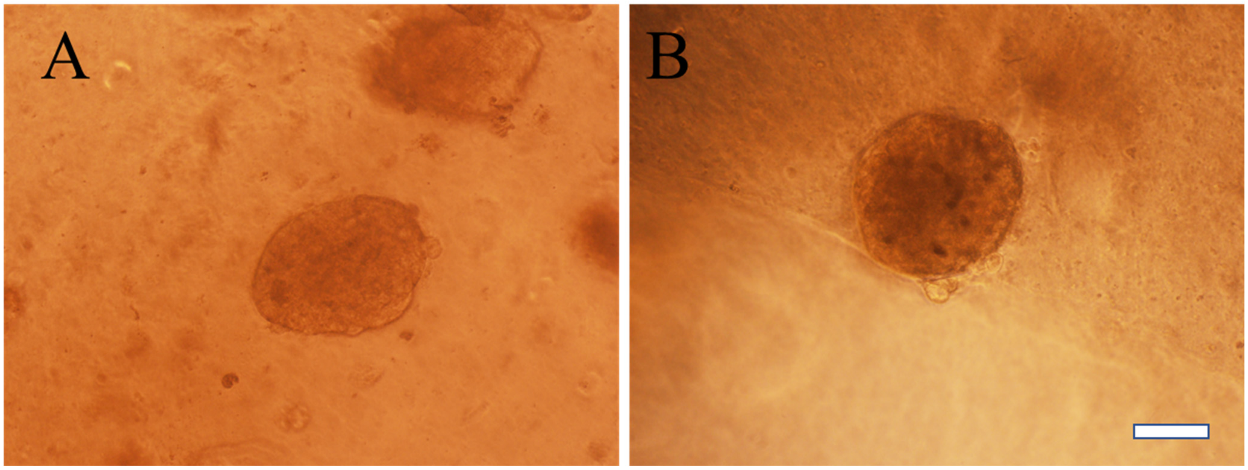

Figure S4. Bright-field observation of the individual Hep-G2: HUVEC clusteroids in 5 mg mL^{-1} Matrigel after 21 days of culture in low-serum medium. The bar is 50 µm.

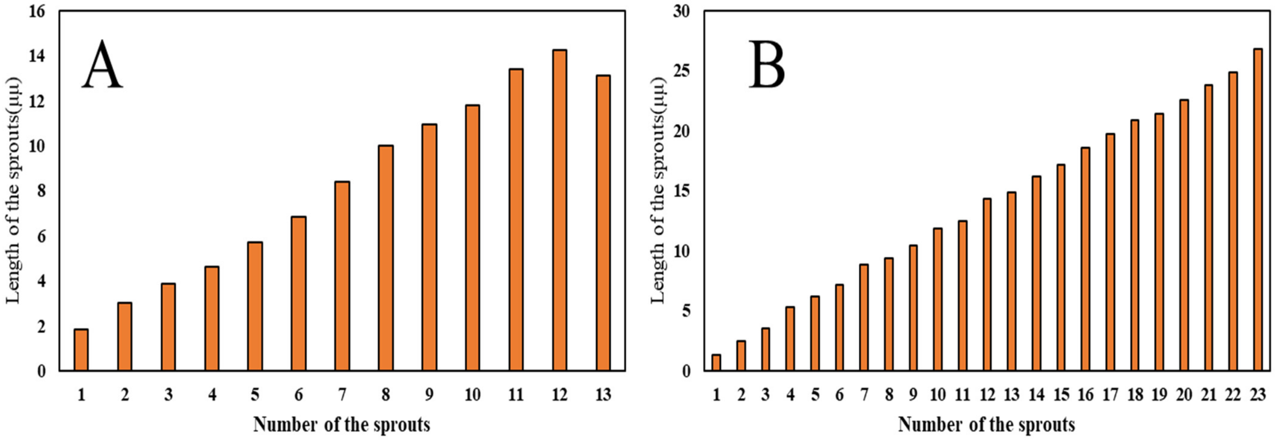

Figure S5. The length of the sprouts in Table S1.

Table S1. The effect of the angiogenesis-related genes on the vascularization process. (+) represents that they are pro-angiogenesis.

**(+)**	**Angiogenin**
(+)	angiopoietin-2
(+)	HB-EGF
(+)	IGFBP-1
(+)	IGFBP-3
(+)	MMP-9
(+)	Pentraxin 3 (PTX3)
(-)	Serpin E1
(-)	Serpin F1
(-)	TIMP-1
(-)	TIMP-4
(+)	Thrombospondin-1
(+)	uPA
(+)	VEGF
